# Supplementary material for: Caspr1 is a host receptor for meningitis-causing Escherichia coli
Source: Nat Commun. 2018 Jun 12;9:2296. doi: 10.1038/s41467-018-04637-3 (PMC5997682; doi:10.1038/s41467-018-04637-3)
Supplement: Supplementary file 1 — Supplementary Information [file 41467_2018_4637_MOESM1_ESM.pdf]

Supplementary information

Caspr1 is a host receptor for meningitis-causing *Escherichia coli*

Zhao et al.

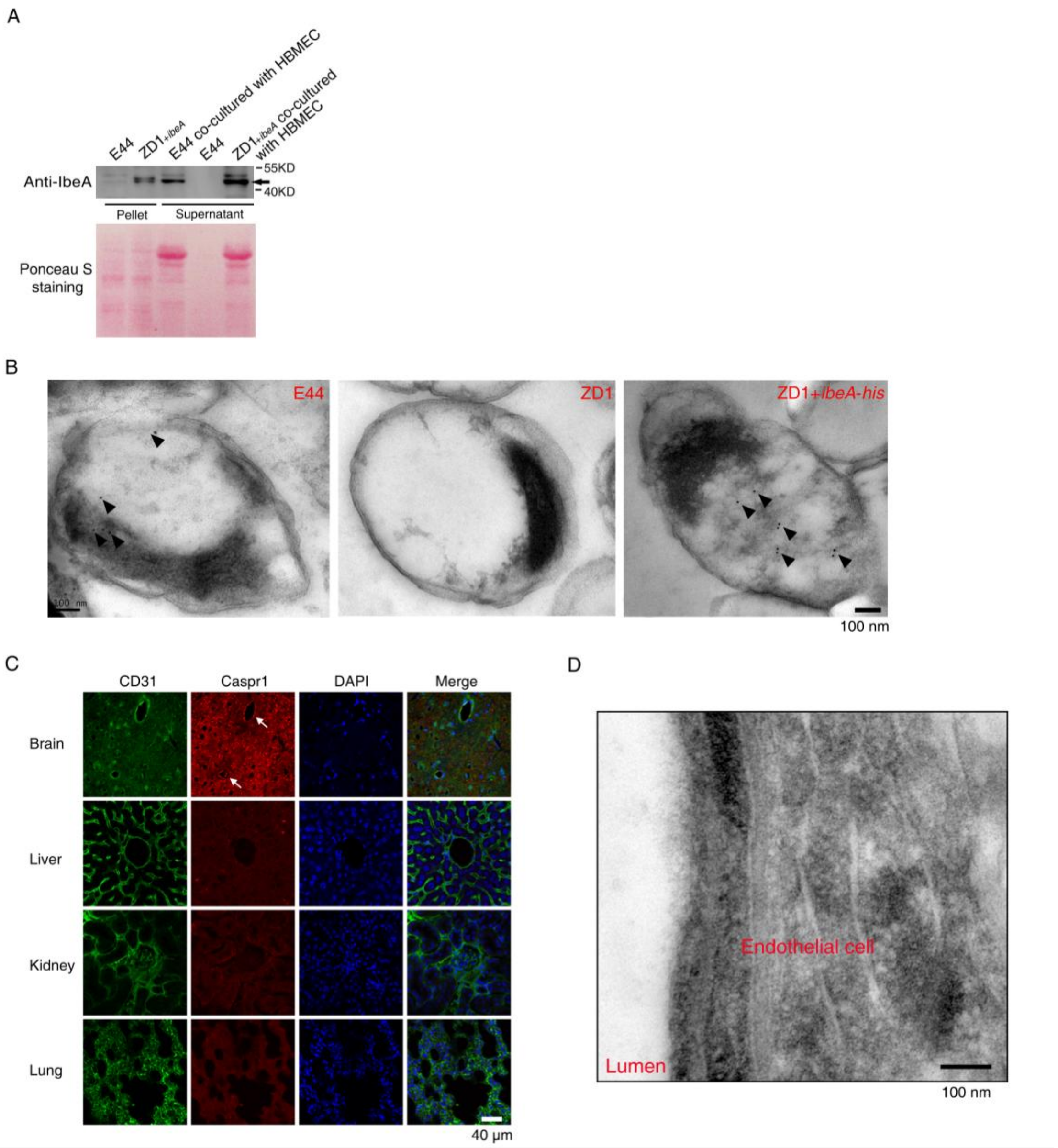

Supplementary Figure 1 Related to Figure 1. Characterization of IbeA secretion and Caspr1 expression

(A) *E. coli* was cultured overnight and the bacterial pellet was collected and lysed for Western blot analysis with antibody recognizing IbeA (the left 2 lanes). When indicated, the overnight cultured *E. coli* was co-cultured with HBMEC for 15 min and the supernatant were collected for Western blot with IbeA antibody (the right 3 lanes). Note that the supernatant of wild-type *E. coli* (E44) cultured alone for 15 min (without co-culture with HBMECs) was used as control (the second lane from the right). E44, wild-type *E. coli*; ZD1, *ibeA*-deleted *E. coli*; ZD1$_{+ibeA}$, ZD1 transformed with full-length *ibeA* gene.

(B) The ultrathin sections of wild type *E. coli* (E44), *ibeA*-deleted ZD1 strain, and ZD1 transformed with constructs encoding his-tagged *ibeA* were prepared. Then the sections were blocked with 5 % donkey serum in PBS at room temperature, and then incubated with anti-IbeA monoclonal antibody followed by donkey anti-mouse IgG coupled to 10-nm gold particles. Representative electron microscope micrographs from 3 independent experiments were presented. Arrowheads indicate gold particles inside the bacterial cytosol.

(C) The slices of rat tissues were obtained and stained with mouse anti-CD31 and rabbit anti-Caspr1 antibodies. Then the slices were incubated with Alexa Fluor 488-conjugated anti-mouse IgG (Green) and Alexa Fluor 594-conjugated anti-rabbit IgG (Red). DAPI was used to stain the nucleus (blue). Samples were analyzed using confocal microscopy (FluoView FV1000, Olympus). Arrows indicate the positive staining of Caspr1 in brain microvessels. Images are representative of three independent experiments.

(D) Ultrathin sections were prepared from the rat brain and stained with isotype IgG (as control), followed by incubation with secondary antibody conjugated with 10-nm gold particles. Images were acquired using transmission electron microscope. Lumen indicates the vascular lumen. Images are representative of three independent experiments.

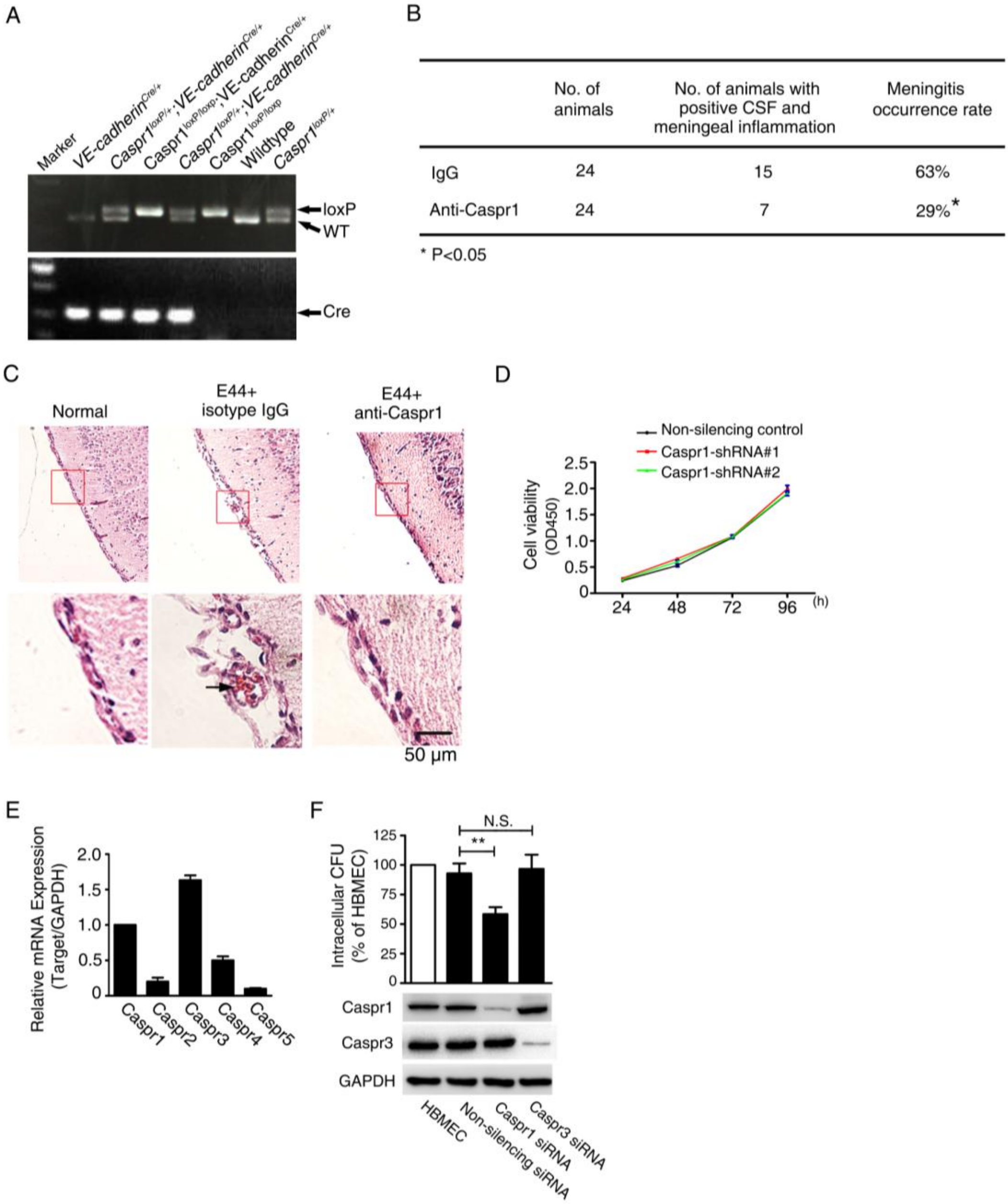

Supplementary Figure 2 Related to Figure 2. Caspr1 is associated with bacterial penetration through the BBB in *E. coli* meningitis

(A) *Caspr1*$^{loxP/loxP}$ mice were crossed with VE-cadherin-Cre mice in which the codon-improved Cre recombinase gene is expressed under the control of the promoter of the *VE-cadherin* gene. Then *Caspr1*$^{loxp/+}$*; VE-cadherin*$^{Cre/+}$ mice were self-crossed to obtain *Caspr1*$^{loxP/loxP}$*; VE-cadherin*$^{Cre/+}$ mice with endothelial-specific knockout of Caspr1. The genotype of mice was identified by PCR genotyping. The primer sequence of loxP was CTGGGCACTAGCCTGAAGGGAAG (forward) and

GAACAGTAGTAACCATGTCCCCACCC (reverse). The primer sequence of Cre recombinase was CCTGTTTTGCACGTTCACCG (forward) and ATGCTTCTGTCCGTTTGCCG (reverse).

(B-C) The antibody recognizing extracellular region of Caspr1 (200 μl/rat, 0.2 μg/μl) were injected intraperitoneally into neonatal rats, with isotype IgG as negative control. After 3 h, 1 x 10^5 wild-type *E. coli* was injected subcutaneously into the rats. At 18 h post-infection, the blood samples were collected for bacteremia measurement. In the meantime, the CSF were collected and cultured to indicate the passage of bacteria through the BBB. (C) Histological examination of the brain sections was performed to assess the inflammation of the cerebral meninges indicated as meningeal thickening and neutrophils infiltration. The representative images from 3 independent experiments were provided. The lower panels are a higher magnification of the boxed regions in the upper panels. Arrows indicate neutrophils. The rate of meningitis occurrence (B) was calculated by dividing the number of rats with both positive CSF culture and meningeal inflammation by the total number of rats. *, $P<0.05$ (Chi-square).

(D) Stable HBMECs cell lines with Caspr1 knocked down were seeded in 96-well plate and cultured at indicated times. Then the cells were subjected to cell viability assay with cell proliferation reagent WST-1. Values are mean ± SD from 3 independent experiment.

(E) The total RNA of HBMECs was extracted and reverse transcribed using Moloney murine leukemia virus (M-MLV) reverse transcriptase. Then Real-time PCR was performed to detect the mRNA expression levels of Caspr family members (from Caspr1 to Caspr5) in HBMECs, with GAPDH as an internal control. Data are normalized to Caspr1 which was defined as 1. Values are mean ± SD from 3 independent experiment.

(F) HBMECs were transiently transfected with siRNA against Caspr1 and Caspr3, respectively, with non-silencing siRNA as control. The expression levels of Caspr1 and Caspr3 were analyzed by Western blot (lower panel). The transfected HBMECs were used for bacterial invasion assay (upper panel). Values are mean ± SD from 3

independent experiment. **, $P<0.01$. The absolute value of the intracellular bacterial CFU in control (HBMECs) was 3.1 ±1.4 x 10^3 CFU/well.

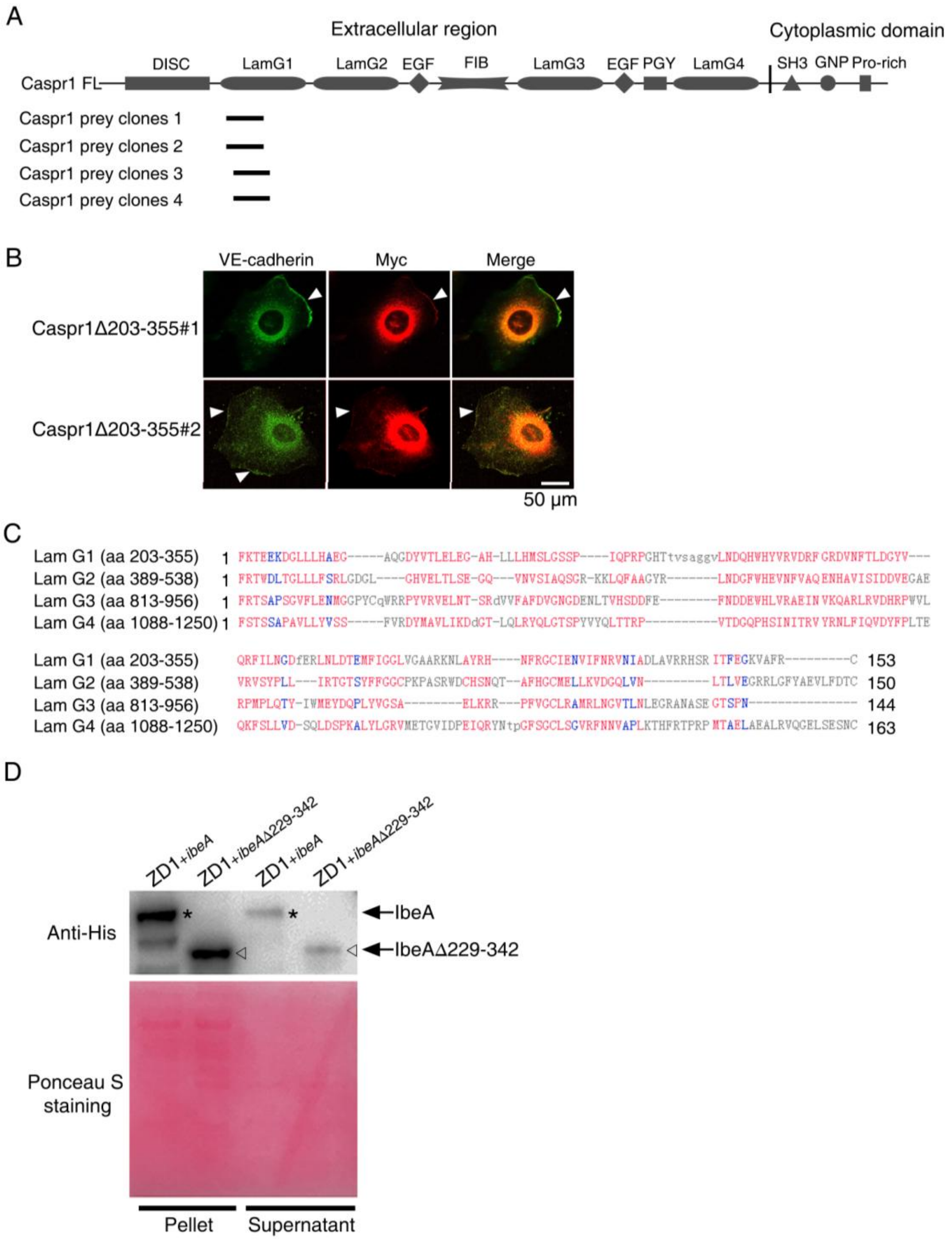

Supplementary Figure 3 Related to Figure 3. Further characterization of the interacting domain in Caspr1(203-355) and the secretion of IbeAΔ229-342 from bacteria.

(A) Sequence alignment analysis using the translated DNA sequencing results of the positive clones interacted with IbeA in yeast two-hybrid (Y2H) screen revealed that these 4 clones corresponded to the N-terminal laminin-globular (lamG) domain of

Caspr1. These 4 Caspr1 prey fragments in the positive clones from Y2H screen were marked as lines aligned with the schematic Caspr1 domain structure (FL, full-length).

(B) HBMECs were stably transfected with Caspr1 mutant with deletion of aa 203-355 (Caspr1Δ203-355), and the expression of mutated Caspr1Δ203-355 (with Myc tag) were stained with antibodies recognizing Myc (Red), and VE-cadherin (Green) was stained as a marker for plasma membrane. Arrowheads indicate the co-localization of Caspr1Δ203-355 with VE-cadherin at the plasma membrane. Images are representative of 3 independent experiments.

(C) Multiple sequence alignment of the 4 lam G domains in Caspr1, including lam G1 (aa 203-355), lam G2 (aa 389-538), lam G3 (aa 8133-956) and lam G4 (aa 1088-1250). The red font indicates the highly conserved amino acids and the blue font indicates less conserved ones, whereas the grey ones indicate the amino acids without any homology. The lower case letters indicate the unique amino acids that are not found in the other 3 lam G domains.

(D) The *ibeA*-deleted ZD1 strain was transfected with full-length *ibeA* and *ibeAΔ229-342* containing His-tag, respectively. Then the *E. coli* were co-cultured with HBMECs and the supernatant were collected for Western blot analysis with anti-His antibody. The bacterial pellet of the *E. coli* without co-incubation with HBMECs were loaded as positive controls. The position of IbeA and IbeAΔ229-342 proteins were marked on the right of the image. Images are representative of 3 independent experiments.

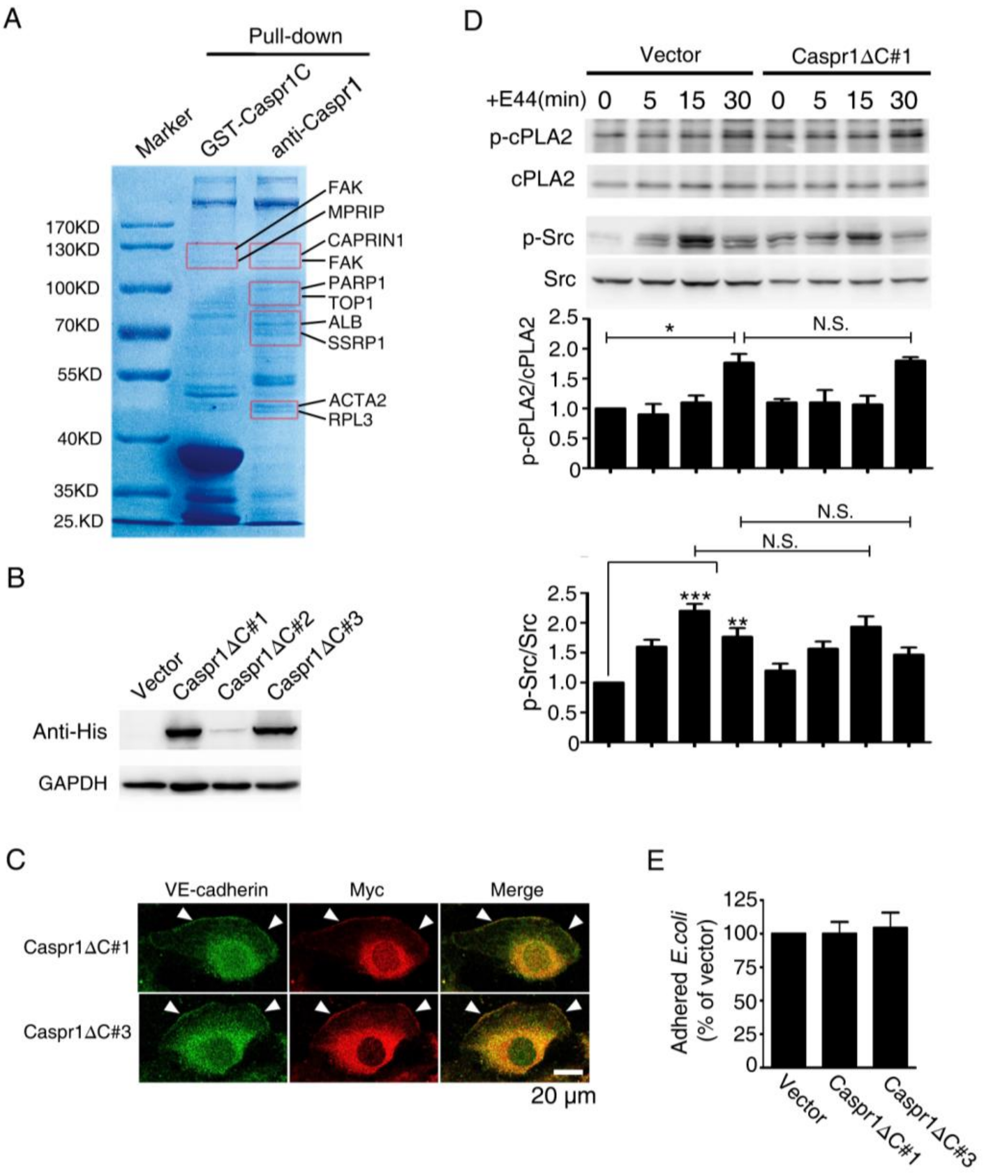

Supplementary Figure 4 Related to Figure 4. Identification of the intracellular signaling pathways associated with Caspr1 in HBMECs.

(A) The cell lysate of HBMECs was incubated with the GST-tagged cytoplasmic tail of Caspr1 (GST-Caspr1C) and Caspr1 antibody (anti-Caspr1), respectively. Then the lysate was precipitated with Glutathione Sepharose beads and protein A/G beads, respectively. The precipitated proteins were subjected to SDS-PAGE followed by Coomassie brilliant blue staining. The gel bands (labeled by the red box) were cut and analyzed by mass spectrometry. The identified precipitated proteins were labeled on the right. FAK, focal adhesion kinase; MPRIP, myosin phosphatase Rho-interacting protein; CAPRIN1, cell cycle associated protein 1; PARP1, poly (ADP-ribose)

polymerase 1; TOP1, topoisomerase (DNA) I; ALB, albumin; SSRP1, structure specific recognition protein 1; ACTA2, actin alpha 2; RPL3, ribosomal protein L3.

(B) HBMECs were stably transfected with the construct encoding Caspr1 with deletion of cytoplasmic tail (Caspr1ΔC), with the empty vector served as control. The expression of Caspr1ΔC (with His tag) in the stably transfected HBMECs was identified by Western blot, with GAPDH as loading control.

(C) HBMECs were stably transfected with Caspr1ΔC mutant construct, and the expression of mutated Caspr1ΔC (with Myc tag) were stained with antibodies recognizing Myc (Red). VE-cadherin (Green) was stained to mark the plasma membrane. Arrowheads indicate the co-localization of Caspr1ΔC with VE-cadherin at the plasma membrane. Images are representative of 3 independent experiments.

(D) HBMECs stably transfected with Caspr1ΔC were infected with wild-type *E. coli* (E44) for indicated times. Then the cells were lysed and the phosphorylation of cPLA2 and Src was analyzed by Western blot using antibodies against cPLA2 and p-cPLA2 (phospho-cPLA2), Src and p-Src (phospho-Src), respectively. HBMECs stably transfected with empty vector served as control. The densitometric quantification of the bands in Western blot were performed and the statistical significance were labeled. Values are mean ± SD from 3 independent experiment. *, $P<0.05$; **, $P<0.01$; ***, $P<0.001$.

(E) HBMECs stably transfected with Caspr1ΔC were subjected to bacterial adhesion assay, with HBMECs stably transfected with vector as a control. Data are presented as relative adhesion compared to control, defined as 100%. Values are means ± SD of 3 independent experiments done in triplicate.

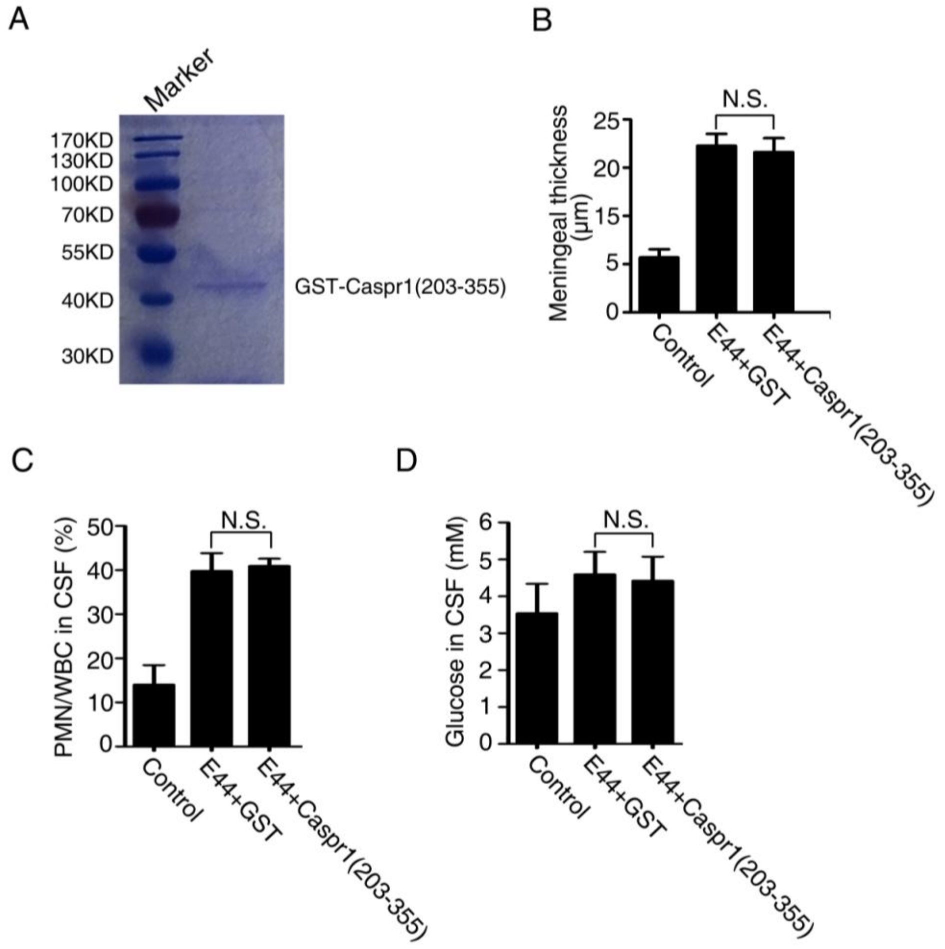

Supplementary Figure 5 Related to Figure 5. Purification of Caspr1(203-355) recombinant proteins and the effect of Caspr1(203-355) on *E. coli* meningitis.

(A) The coding sequence of Caspr1(203-355) were subcloned into pFastBac1 containing GST tag and transfected into Sf9 insect cells. The Sf9 cells expressing GST-tagged Caspr1(203-355) were harvested by centrifugation, then the supernatant was loaded into Glutathione-Sepharose 4B column for purification. The GST-Caspr1(203-355) protein was eluted and subjected to SDS-PAGE followed by Coomassie brilliant blue staining.

(B-D) Further analysis of the rats with meningitis in Fig. 5D. In brief, the neonatal rats were subcutaneously inoculated with 1 x 10^5 CFUs of E44 pretreated with GST-Caspr1(203-355) peptide (30 μg/rat), with GST protein as control. At 18 h post-infection, all the rats with meningitis identified as positive CSF culture in Fig. 5D, were included for additional analysis as follows: (B) The brains were harvested and sectioned for hematoxylin and eosin (HE) staining and the meningeal thickness

reflecting the degree of meningitis were measured. (C) The ratio of polymorphonuclear (PMN) dividing white blood cells (PMN/WBC) in the CSF were calculated, and (D) the glucose concentrations in CSF were measured. Data are means ± SD. n=14 for E44+GST group (from Fig. 5D), n=6 for E44+Caspr1(203-355) group (from Fig. 5D).

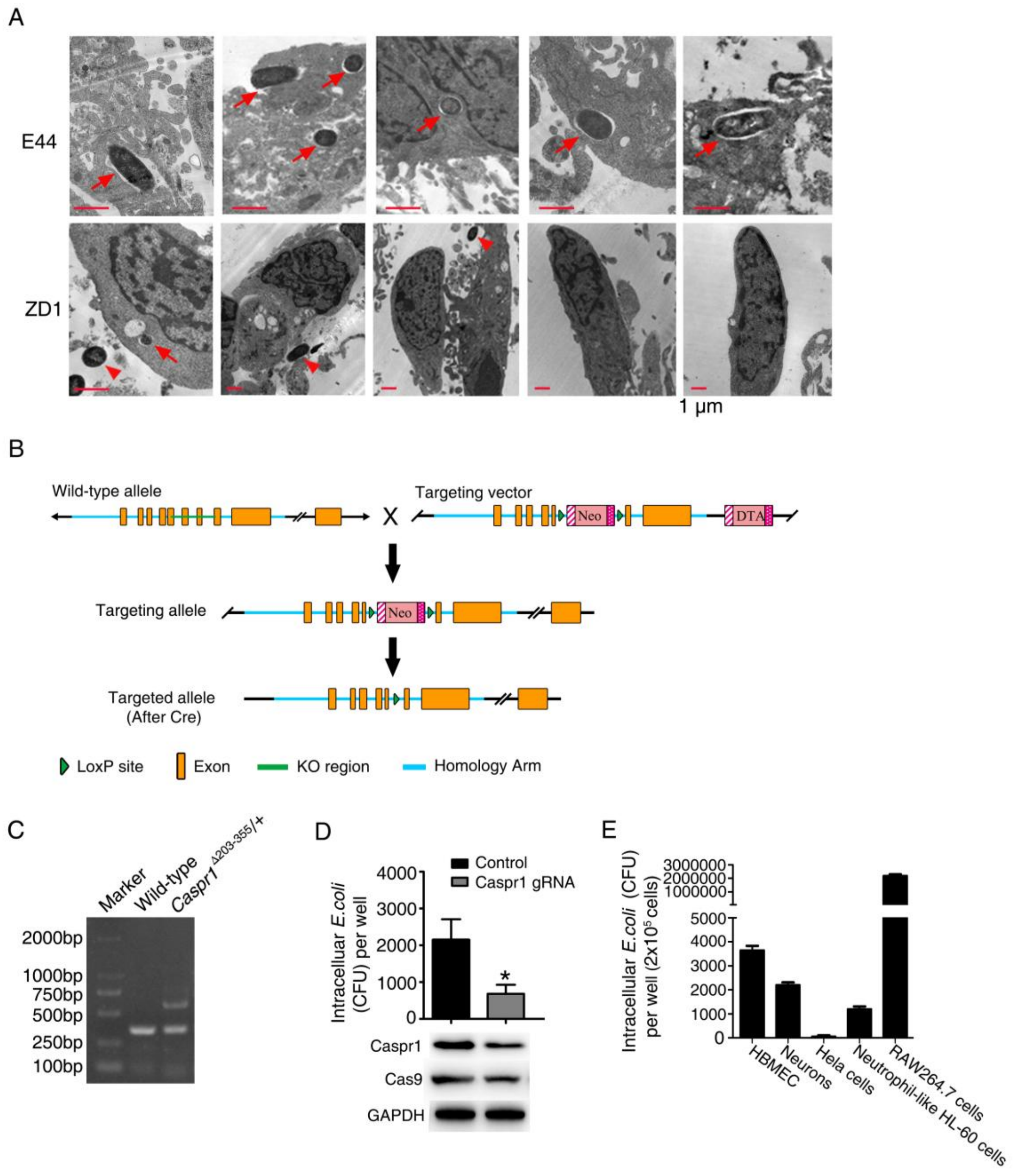

Supplementary Figure 6 Related to Figure 6. *E. coli* is able to invade into neurons, dependent on neuronal Caspr1 and bacterial IbeA

(A) Primary cultured rat hippocampal neurons were infected with wild-type *E. coli* (E44), and *ibeA*-deleted ZD1 strain, respectively. Then the cells were fixed and subjected to transmission electron microscopy analysis. Arrows indicate *E. coli* body inside the neurons; arrowheads indicate *E. coli* outside of the neurons. The representative images are from 3 independent experiment.

(B) Gene-targeting strategy for the generation of transgenic mice harboring deletion of 203-355 domain in Caspr1 with Cre-loxP recombination system. Two loxP sites

were inserted between exon 5 and exon 8 of *Caspr1* gene, respectively. The knockout region in Caspr1 is corresponding to lamG1, i.e., the 203-355 domain, and the reading frame of *Caspr1* gene remained unaffected. The obtained heterozygous transgenic mice were name as Caspr1$^{\Delta 203\text{-}355/+}$.

(C) PCR genotyping results of the Caspr1$^{\Delta 203\text{-}355/+}$ transgenic mice, with wild-type mice as a control. The sequence of the forward primers was GGCTCGCTACATCCGCATC. The sequence of the two reverse primers were TACATTCACTTGCCCTTCGCTA and GCGCCCCTTGCAGTTCAA.

(D) Primary rat hippocampal neurons were cultured and the neuronal Caspr1 were down-regulated by CRISPR-cas9 technique. The downregulation of Caspr1 was identified by Western blot (bottom panel). Then *E. coli* invasion assay were conducted with Caspr1-downregulated neurons. Values are mean ± SD of four independent experiments. *, P<0.05. The absolute value of the control in bacterial invasion assay was 2.14 ± 1.03 x 10^3 CFU/well.

(E) Bacterial invasion assays were performed with several different types of cells, including HBMECs, primary rat hippocampal neurons, cervical cancer HeLa cells, neutrophil-like HL-60 cells (differentiated myeloid leukemia cells induced by dimethylsulfoxide), murine macrophages RAW264.7 cells. The Y-axis was defined as invaded *E. coli* (CFU) per well (with the number of host cells indicated) to compare the invasion efficiency. Values are mean ± SD of 3 independent experiments.

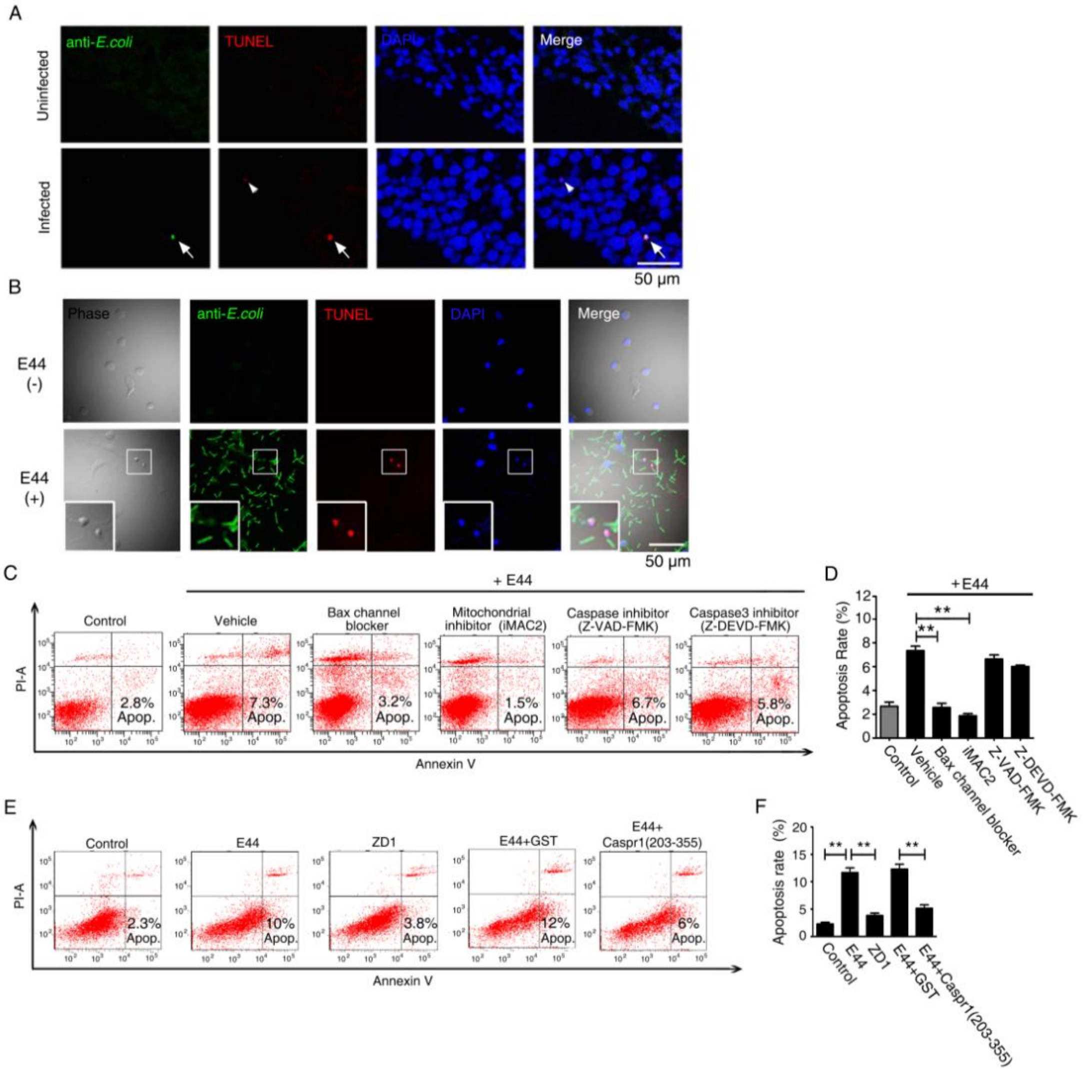

Supplementary Figure 7 Related to Figure 6. *E. coli* infection resulted in neuronal apoptosis, dependent on IbeA-Caspr1 interaction.

(A) Neonatal rats were infected with wild-type *E. coli* K1 and the brains of rat with positive CSF (containing penetrated bacteria) were sectioned and used for TUNEL staining (red). The bacteria were stained with *E. coli* antibody (green). Arrow indicate the apoptotic neurons co-localized with *E. coli* in the brain parenchyma. Arrowhead indicate neuronal apoptosis without recognizable associated *E. coli*. Scale, 50 μm. Images are representative of three independent experiments.

(B) Primary cultured rat hippocampal neurons were infected with *E. coli* (E44) for 30 min and then the cells were subjected to TUNEL staining (red). *E. coli* were stained with anti-*E. coli* antibody (green). Scale, 50 μm. Images are representative of three independent experiments.

(C and D) Primary cultured rat hippocampal neurons were infected with *E. coli* K1 for 30 min followed by the staining with Annexin V-FITC and propidium iodide (PI). The rate of cell apoptosis was determined by flow cytometry analysis, which was labeled on the lower right quadrant. When indicated, the neurons were pretreated with apoptosis inhibitors including Bax channel blocker (10 μM, Tocris), mitochondrial inhibitor iMAC2 (10 μM, Tocris), pan Caspase inhibitor Z-VAD-FMK (10 μM, Tocris) and Caspase3 inhibitor Z-DEVD-FMK (10 μM, Tocris), respectively, for 30 min before bacterial infection. Images are representative of 3 independent experiments. n=3. **, P<0.01 (one way ANOVA).

(E and F) Primary cultured hippocampal neurons were incubated with *E. coli* for 30 min and then the cells were stained with Annexin V-FITC and propidium iodide (PI). The rate of cell apoptosis was determined by flow cytometry analysis, which was labeled on the lower right quadrant. The ability of E44 and ZD1 strain to induce neuronal apoptosis was measured and compared. When indicated, the neurons were pretreated with Caspr1(203-355) peptides, with GST as a control. Images are representative of three independent experiments. n=3. **, P<0.01 (one way ANOVA).

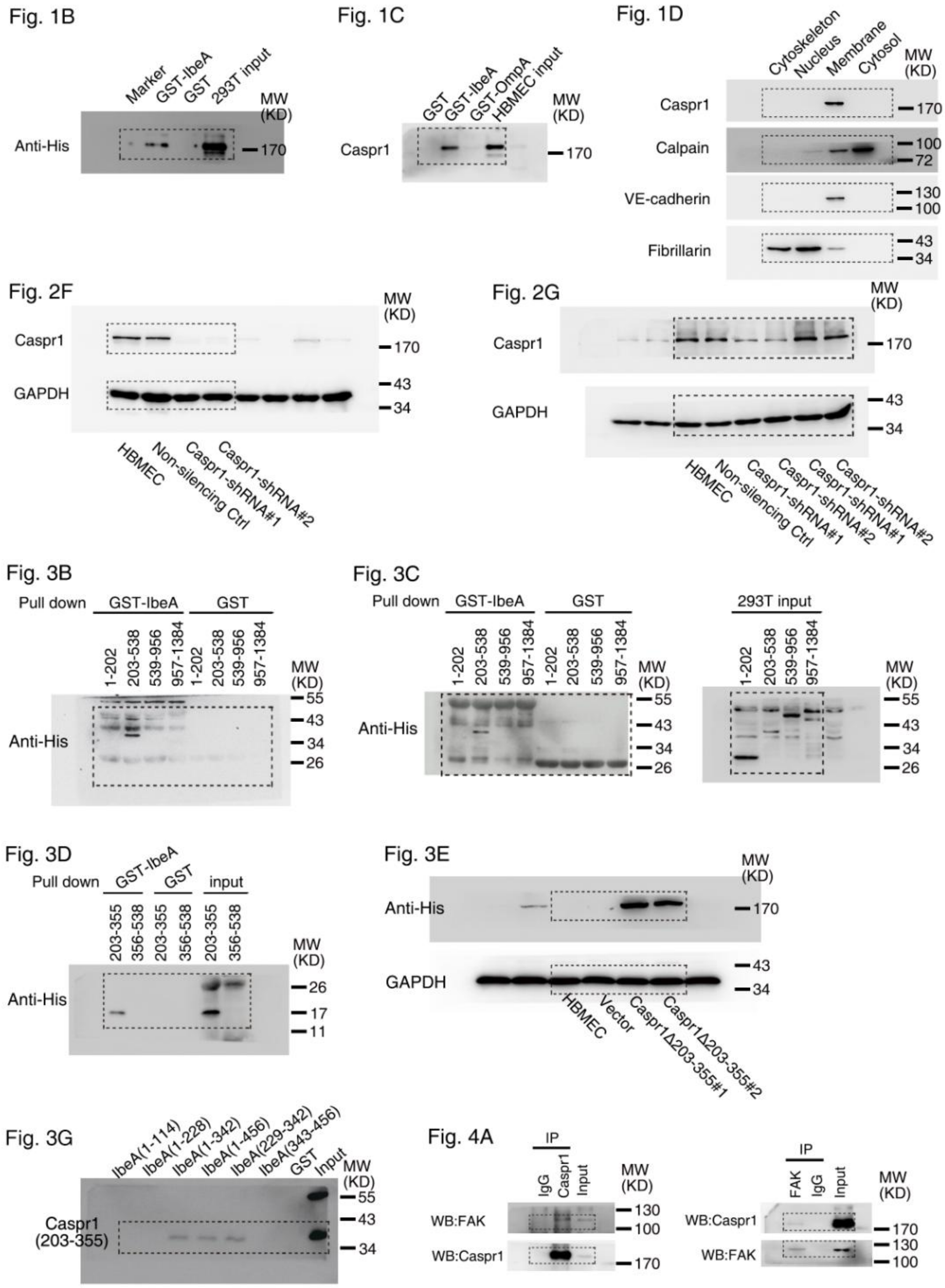

Supplementary Figure 8. Raw data of Immunoblotting from all Figures.

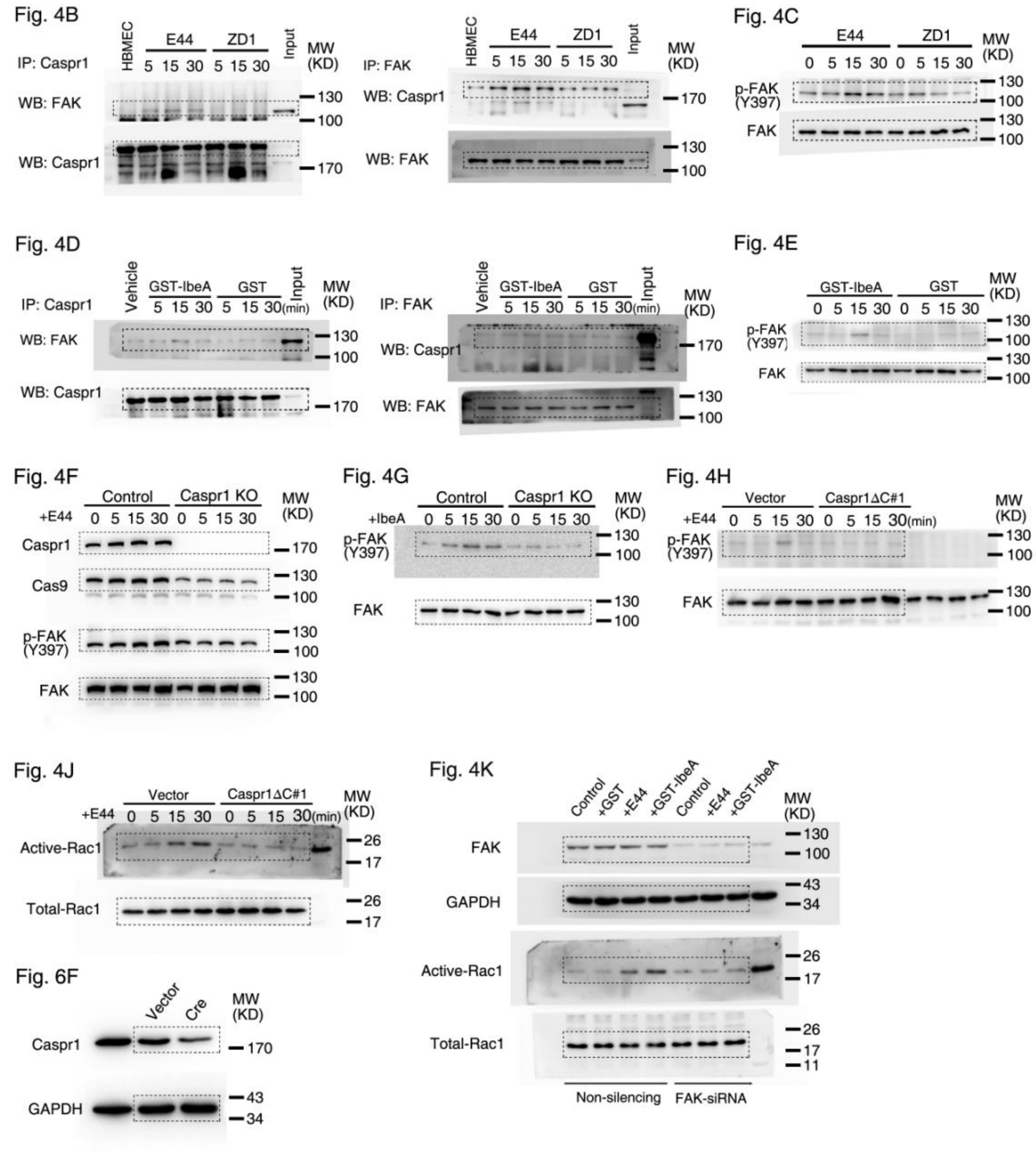

Supplementary Figure 8. Raw data of Immunoblotting from all Figures.

Supplementary Table 1 Results of yeast two-hybrid screen using IbeA as bait protein

Clone #	Genbank accession#	Gene name
35, 66	NM_001101	Actin, beta
10	NM_004077	Citrate synthase
40, 62, 65, 72	NM_003632	Caspr1 (contactin associated protein 1)
2, 6, 20, 22, 24, 34, 50, 61	BC065494	DEAD/H (Asp-Glu-Ala-Asp/His) box polypeptide 12
19, 46, 47, 58	NM_000402	Glucose-6-phosphate dehydrogenase
33	NM_007260	Lysophospholipase II
3, 56	NM_015889	Mediator complex subunit 15
11	AY129569	Polymerase (DNA directed), delta 1, catalytic subunit
32	NM_001105570	Nudix hydrolase 19
4, 8, 15, 25, 26, 27, 38, 39, 67, 68, 69	NM_020850	RAN binding protein 10
14, 63	NM_133452	Ribonucleoprotein, PTB binding 1
29, 57	BC013878	Thimet oligopeptidase 1
41	NM_006086	Tubulin beta 3 class III
5	NM_004651	Ubiquitin specific peptidase 11

Supplementary Table 2 Constructs containing different forms of human Caspr1

Amplicon	Primer sequence, 5'-3', forward, reverse	Restriction enzyme	Clone vector	Purpose
aa 1-1384 (full length)	CCG***GAATTC***CACCATGATGCATCTCCGGCTCTTCTG,<br>TGC***TCTAGA***CCTTCAGACCTGGACTCCTCCAGGAT	*EcoRI*, *XbaI*	pcDNA3.1myc/his B	GST pull-down
aa 1-202	CCG***GAATTC***CACCATGATGCATCTCCGGCTCTTCTG,<br>GC***TCTAGA***CTGCTGAAGGCGAACAC	*EcoRI*, *XbaI*	pcDNA3.1myc/his B	GST pull-down
aa 203-538	CCG***GAATTC***CACCATGTTCAAGACCGAGGAGAAGG,<br>TGC***TCTAGA***CTACATGTATCAAAGAGGACCTCAG	*EcoRI*, *XbaI*	pcDNA3.1myc/his B	GST pull-down
aa 539-956	CCG***GAATTC***CACCATGGGCATCACTGATAGGTGC,<br>TGC***TCTAGA***CCGTTGGGTGAGGTACCCTCAG	*EcoRI*, *XbaI*	pcDNA3.1myc/his B	GST pull-down
aa 957-1384	CCG***GAATTC***CACCATGTGCACAGGCCACTGTGC,<br>TGC***TCTAGA***CCTTCAGACCTGGACTCCTCCAG	*EcoRI*, *XbaI*	pcDNA3.1myc/his B	GST pull-down
aa 203-355	CCG***GAATTC***CACCATGTTCAAGACCGAGGAGAAGG,<br>GC ***TCTAGA***CTGCAACGAAAAGCCACCTTAC	*EcoRI*, *XbaI*	pcDNA3.1myc/his B	GST pull-down
aa 356-538	CCG***GAATTC***CACCATGCTGGACCCGGTACCGCAC,<br>TGC***TCTAGA***CTACATGTATCAAAGAGGACCTCAG	*EcoRI*, *XbaI*	pcDNA3.1myc/his B	GST pull-down
aa 1-202<br>aa 356-1384	CCG***GAATTC***CACCATGATGCATCTCCGGCTCTTCTG,<br>GGTGCGGTACCGGGTCCAGGCTGAAGGCGAACACGTCCC<br>GGGACGTGTTCGCCTTCAGCCTGGACCCGGTACCGCACC,<br>TGC***TCTAGA***CCTTCAGACCTGGACTCCTCCAGGAT	*EcoRI*, *XbaI*	pcDNA3.1myc/his B	aa 203-355 deletion mutant

aa 1-1304	CCG***GAATTC***CACCATGATGCATCTCCGGCTCTTCTG,<br>TGC***TCTAGA***CCTCTAGAGAGAAGAGCACCAACATTCCCAC	*EcoRI, XbaI*	pcDNA3.1myc/his B	Stable transfection
aa 203-355	CCG***GAATTC***CACCATGTTCAAGACCGAGGAGAAGG,<br>GC ***AAGCTT***CTGCAACGAAAAGCCACCTTAC	*EcoRI, HindIII*	pFastBac1	Recombinant protein expression

Supplementary Table 3 Primers for Real-time RT-PCR in HBMECs

Target gene	Amplicon length (bp)	Primer sequence, 5'-3', forward, reverse	Nucleotide position	Genbank accession No.
Caspr1	100	GATGAAGGATGGGTTGCCATA,<br>AGCGATGATTTTGCAGATAGAAGA	4048-4068<br>4124-4147	NM_003632.2
Caspr2	116	TGCCTAGAGAGATACCACGGTTACT,<br>TTATATCGTAGCCACATCCCTTCTT	3454-3478<br>3545-3569	NM_014141.5
Caspr3	101	CCCTTTGAAGCCAGGTTCCT,<br>TCAGATTTATATGCACATCCGTACAC	684-703<br>759-784	NM_033655.3
Caspr4	101	GTGATGCTGACCGGAATGAA,<br>CAGTCGGCCTGTGTCTGTAATC	2601-2620<br>2680-2701	NM_033401.3
Caspr5	98	TCACTGATACCGACAGATCAAACTC,<br>ATAAAATGAGACGGCGTTCCA	2655-2679<br>2732-2752	NM_130773.3
GAPDH	102	AAGGTGAAGGTCGGAGTCAAC,<br>GGGGTCATTGATGGCAACAATA	195-215<br>275-296	NM_002046.5

Supplementary Table 4 Constructs containing different forms of *E. coli* K1 IbeA

Amplicon	Primer sequence, 5'-3', forward, reverse	Restriction enzyme	Clone vector	Purpose
aa 1-456 (full length)	CCG***GAATTC***CACCATGGAATTTTATCTGGAACC, ACGC***GTCGAC***TTAAAAGACTTTTACGCCATT	*EcoRI, SalI*	pGBKT7	Yeast two-hybrid
aa 1-456 (full length)	GTTTAACTTTAAGAAGGAGATATA***CCATGG***CACCACACCACCACCAC CACCACATGGAATTTTATCTGGA, GTGGTGGTGGTGGTGCTCGAGTGCGGCCGC***AAGCTT***CCTTAAAAGA CTTTTACGCCATTT	*NcoI, HindIII*	pET-28a (+)	His-tagged protein purification
aa 1-456 (full length)	CCG***GAATTC***CACCATGGAATTTTATCTGGAACC, ACGC***GTCGAC***TTAAAAGACTTTTACGCCATT	*EcoRI, SalI*	pGEX4T-3	GST-tagged protein purification
aa 1-114	CCG***GAATTC***CACCATGGAATTTTATCTGGAACC, ACGC***GTCGAC***CATTGCATCCGCAACCAG	*EcoRI, SalI*	pGEX4T-3	GST-tagged protein purification
aa 1-228	CCG***GAATTC***CACCATGGAATTTTATCTGGAACC, ACGC***GTCGAC***TTCATGAACATCATAAGACCAG	*EcoRI, SalI*	pGEX4T-3	GST-tagged protein purification
aa 1-342	CCG***GAATTC***CACCATGGAATTTTATCTGGAACC, ACGC***GTCGAC***TTCCGTAAGCTGGACTCGC	*EcoRI, SalI*	pGEX4T-3	GST-tagged protein purification
aa 229-342	CCG***GAATTC***TCTTGTCGCGATATGTTTAGC, ACGC***GTCGAC***TTCCGTAAGCTGGACTCGC	*EcoRI, SalI*	pGEX4T-3	GST-tagged protein purification

aa 343-456	CCG***GAATTC***AATGATATTTGTAATGAGGGAC, ACGC***GTCGAC***TTAAAAGACTTTTACGCCATT	*EcoRI, SalI*	pGEX4T-3	GST-tagged protein purification
aa 1-456 (full length)	ACGC***GTCGAC***ATGGAATTTTATCTGGAACC, CCG***GAATTC***TTAAAAGACTTTTACGCCATT	*SalI, EcoRI*	pUC13	Complementation analysis
aa 1-228<br>aa 343-456	ACGC***GTCGAC***ATGGAATTTTATCTGGAACC, TTCATGAACATCATAAGACCAG<br>CTGGTCTTATGATGTTCATGAAAATGATATTTGTAATGAGGGAC, CCG***GAATTC***TTAAAAGACTTTTACGCCATT	*SalI, EcoRI*	pUC13	aa 229-342 deletion mutant of IbeA
aa 1-228<br>aa 343-456	ACGC***GTCGAC***GGAGAAGGTATTTATTAACTGCAA, TTCATGAACATCATAAGACCAG<br>CTGGTCTTATGATGTTCATGAAAATGATATTTGTAATGAGGGACA, TTC***GAGCTC***AGGTAAAGCACAGAACAATAAG	SalI, SacI	pCVD442	Construction of *IbeA*Δ229-342 isogenic in-frame deletion mutant strain
